# Family physician versus specialist family physicians: Curse or edge of collapse?

**DOI:** 10.4102/phcfm.v18i1.5503

**Published:** 2026-06-25

**Authors:** Prosper M. Lutala, Jessie Z. Mbamba, Patrick W. Chisepo, Anna C. McDonald, Modai Mnenula

**Affiliations:** 1 Department of Family Medicine, School of Medicine and Oral Health, Kamuzu University of Health Sciences, Blantyre, Malawi; 2 Swedish Family Medicine First Hill, Department of Family Medicine, University of Washington, Seattle, Washington, United States of America

**Keywords:** family physician, specialist family medicine, minor ailments consultation, undifferentiated, continuity

## Abstract

The introduction and growth of family medicine as a specialty in Africa have produced mixed outcomes. While the consolidation of training has enhanced credibility, academic rigour, and career pathways, it has also contributed to a decline in the management of undifferentiated and complex cases, which form the bulk of primary care encounters. Increasingly, these cases are categorised as ‘minor ailments’, leading to reduced engagement of specialists with patients, communities, and the foundational principles of the discipline. This trend risks distancing family medicine from its holistic, community-oriented roots. Beyond qualification titles, the discipline must actively seek ways to sustain its presence in routine consultations, ensuring that specialists remain engaged in everyday patient encounters while applying advanced skills to address complexity.

## Introduction

### Preserving the Spirit of Family Medicine: Lessons from Early Leaders

In remembrance of Prof. George Samuel (Sam) Fehrsen (26 August 1938–22 May 2018), written on the seventh anniversary of his passing, 22 May 2025. Seven years after the passing of Prof. Sam Fehrsen, it is fitting not only to recall his leadership in establishing family medicine in Southern Africa, but also to revisit the questions he and his colleagues courageously raised about its future. Fehrsen was not merely a founder of training programmes; he was a guardian of ethos. His concern was never about titles, but about spirit. In 2013, together with colleagues, they warned: ‘We are extremely worried by reports of family physician specialists who consider themselves to be too important to see patients with so-called minor ailments’.^1^ This was not rhetorical exaggeration. It was a caution, perhaps even a prophecy.

## The family physician before specialist recognition

Before formal specialist pathways were widely established, the family physician in many African contexts was defined by scope rather than status. They worked in district hospitals, rural health centres, and community clinics. They delivered babies at night, managed tuberculosis in the morning, counselled families in the afternoon, and admitted critically ill patients when referral was impossible. Their authority came from presence. Primary care scholarship has consistently shown that systems grounded in first-contact, comprehensive, and continuous care achieve better health outcomes and greater equity.^2,3,4^ The ecology of medical care demonstrates that most health problems are resolved at the primary care level.^5^ This was the family physician’s natural habitat. The so-called ‘minor ailments’ were never minor. A headache might conceal meningitis. A cough might signal tuberculosis. Elevated blood pressure might be the first sign of a lifelong chronic disease. To see everything, and to see everyone, was the discipline’s strength.

Couper, Fehrsen, and Hugo understood this deeply. Their vision of family medicine was not about hierarchy; it was about responsibility.

## The rise of the ‘specialist family physician’

From the mid-2000s, family medicine in Africa underwent professional maturation. Structured postgraduate programmes expanded in South Africa, Nigeria, and elsewhere. Recognition of family medicine as a specialist discipline brought legitimacy, academic rigour, and policy voice.^6,7^

This development aligned with global calls to strengthen primary health care (World Health Organization (WHO) 2008) and with World Organization of Family Doctors’ (WONCA’s) definition of family medicine as a discipline requiring specialist training in comprehensive, person-centred care.^8,9^ Specialist recognition was necessary. Without it, family medicine risked marginalisation in hospital-dominated systems. It enabled research growth, curriculum standardisation, and clear career pathways. But professionalisation also introduces new cultural pressures. As Abbott^10^ argues in his sociology of professions, disciplines compete for status and jurisdiction. Titles matter. Hierarchies influence behaviour. The language of ‘specialist’ carries implicit associations of prestige, selectivity, and procedural dominance.

And here lies the tension.

## Prestige drift and the quiet shift

Fehrsen, Couper, and Hugo’s concern was not that family physicians would become better trained. It was that they might become distanced.

When a discipline internalises the prestige norms of tertiary specialities, subtle shifts occur:

- Administrative roles replace clinical presence.
- Referral oversight replaces first-contact care.
- Complexity is redefined as procedural rather than relational.

The danger is not the overt abandonment of primary care. It is a gradual disengagement from the everyday encounter. In African district health systems, which still bear the dual burdens of communicable and non-communicable disease,^11^ the family physician is often the most stable clinical anchor. If that anchor is not strongly rooted in the intimacy of the humanity revealed at each clinical encounter between doctor and patient, the system destabilises. The ‘minor ailment’ becomes symbolic. It represents the ordinary consultation: the rash, the back pain, the insomnia, the routine antenatal visit. If these are seen as beneath the specialist family physician, the discipline’s centre of gravity moves. And when the centre moves, identity follows.

## Specialist generalism: Strength or contradiction?

There is, however, a more hopeful reading. Family medicine has always claimed a form of higher-order expertise: the ability to integrate biomedical, psychological, and social dimensions over time. World Organization of Family Doctors frames the family physician as a specialist in managing complexity within primary care.^9^ If specialist recognition strengthens competence in multimorbidity, undifferentiated diagnosis, continuity across life stages, and system leadership at the district level, then the discipline is strengthened. But if specialist identity encourages withdrawal from first-contact care, the discipline becomes structurally strong and spiritually weak. Fehrsen, Couper, and Hugo’s warning reminds us that collapse, if it comes, will not be dramatic. It will be quiet. It will occur when the family physician no longer sees themselves primarily in the consultation room with undifferentiated illness.

## The African responsibility

Family medicine in Africa carries a particular moral weight. In contexts of workforce scarcity, inequity, and fragile referral systems, the family physician is not simply another specialist. They are a bridge.

Mash et al.^7^ described the African family physician as an expert generalist, capacity builder, consultant, and clinical leader within the district health system. This model presupposes continued clinical engagement. The World Health Report 2008 warned against excessive hospital centrism and called for renewed commitment to primary health care.^8^ Family physicians were meant to be central to that renewal.

If they drift towards hospital identity alone, the reform agenda fractures.

## Remembering Fehrsen’s and acknowledging Couper and Hugo’s spirits

To commemorate Fehrsen and acknowledge Couper and Hugo’s foresight is to interrogate ourselves. They helped build specialist training precisely so that family physicians would be taken seriously. Yet they feared that being taken seriously might tempt them to take themselves too seriously. The spirit they embodied was one of humility, accessibility, and intellectual rigour grounded in service. They understood that the power of family medicine lies in its willingness to remain close to ordinary illness:

- The cough.
- The rash.
- The uncomplicated hypertension review.
- The anxious mother.

These are not beneath the discipline. They are its foundation.

## Conclusion: Edge of collapse or edge of renewal?

Family medicine today stands at a threshold. It has achieved recognition, institutional authority, and academic maturity. These are gains worth protecting. Specialist training has strengthened competence, leadership, and academic credibility. In many African contexts, it has given family physicians the voice they long deserved. But the discipline must constantly ask: *A specialist in what?*

If the answer is ‘specialist’ in terms of relationship, continuity, comprehensiveness, and contextual complexity, then specialist recognition is a strength. If the answer drifts towards hierarchy, distance, and prestige selection, then Couper, Fehrsen and Hugo’s concern^1^ becomes prophetic. The future of family medicine will not be decided in policy documents or curricula alone. It will be decided in consultation rooms whether a family physician chooses to see the patient for the so-called minor ailment. That choice will determine whether the title ‘specialist family physician’ becomes a crown or a quiet sign of erosion.

To young family medicine trainees: You inherit not only a specialist qualification, but a moral tradition. Your expertise will be measured not merely by examinations passed or procedures mastered, but by your willingness to remain present in the ordinary consultation. Do not allow titles to distance you from the undifferentiated patient, the anxious parent, the routine follow-up, or the ‘minor’ ailment that may conceal major suffering. The future of family medicine in Africa will not be secured by prestige, but by proximity, by your decision to stay close to communities, to complexity, and to continuity. Because, in the end, family medicine will not collapse from a lack of status; it will collapse only if it forgets why it exists.
